# Rearing and Biology of *Phlebotomus sergenti*, the Main Vector of Anthroponotic Cutaneous Leishmaniasis in Iran

**Published:** 2017-12-30

**Authors:** Arshad Veysi, Mohamad Reza Yaghoobi-Ershadi, Yavar Rassi, Nasibeh Hosseini-Vasoukolaei, Mahmood Jeddi-Tehrani, Aref Rezaee-Node, Fatemeh Gholampour, Zahra Saeidi, Mahboubeh Fatemi, Mohamad Hossein Arandian, Ali Khamesipour, Amir Ahmad Akhavan

**Affiliations:** 1 Department of Medical Entomology and Vector Control, School of Public Health, Tehran University of Medical Sciences, Tehran, Iran; 2 Department of Medical Entomology and Vector Control, Health Sciences Research Center, Faculty of Health, Mazandaran University of Medical Sciences, Sari, Iran; 3 Monoclonal Antibody Research Center, Avicenna Research Institute, ACECR, Tehran, Iran; 4 International Campus, Tehran University of Medical Sciences, Tehran, Iran; 5 Isfahan Health Research Station, National Institute of Health Research, Tehran University of Medical Sciences, Isfahan, Iran; 6 Center for Research and Training in Skin Diseases and Leprosy, Tehran University of Medical Sciences, Tehran, Iran

**Keywords:** *Phlebotomus sergenti*, Rearing, Biology, Anthroponotic cutaneous leishmaniasis, Iran

## Abstract

**Background:**

Establishment of sand flies laboratory colonies is essential to understand various biological aspects of Phlebotominae sand flies. The aims of the current study were to establish the colony of *Phlebotomus sergenti* Parrot (1917), the main vector of anthroponotic cutaneous leishmaniasis in old world, and to study biological parameters of this species.

**Methods:**

The sand flies were reared at 26–28 °C temperature, 14:10 (light: dark) photoperiod and 70–80% relative humidity. Larval diet was a composted mixture of rabbit faces and rabbit pellets which is prepared through a special process. First to fifth generations of *P. sergenti* were used to define biological parameters.

**Results:**

Results showed that, *P. sergenti* blood feeding percentage were 42% on chicken, 21% on BALB/c and 37% on golden hamster. Average time of blood digestion, egg incubation, 1^st^ instar larva, pupa and adult emerging was recorded at 3.4, 8.7, 15, 33.3 and 41.2 days after blood feeding, respectively. Mean number of laid eggs was 55.1 and retained eggs were 35 per a female. Fecundity and production rate were 61.6%, and 42.2% respectively. Average longevity recorded at 15.2 days for females and 14.8 days for males.

**Conclusion:**

Colony of *P*. *sergenti* has been established for the first time in Iran. Average interval time from egg to adult of this species was 32.5 days. Chicken and golden hamster were recommended as a blood source for colony initiation and routine blood feeding, respectively.

## Introduction

Phlebotomine sand flies (Diptera: Psychodidae) are tiny blood-feeder dipterans which prefer a wide range of hosts for blood feeding, and are potential vectors to transmit a variety of pathogens including bacteria (e.g. *Bartonella bacilliformis*), viruses (e.g. *Phlebovirus*) and protozoa (e.g. *Leishmania* spp) among different host species (1, 2). Leishmaniasis is among neglected diseases and is a major health problem in some endemic regions, according to WHO estimation about a tenth of the world’s population are at risk, and more than 2 million people are infected (3). *Leishmania tropica* is one of the causative agents of Anthroponotic cutaneous leishmaniasis (ACL) and is endemic in many parts of Middle East including Iran (4–6). Recently some studies showed that *L. tropica* CL appeared as zoonotic and was detected in sylvatic cycle (7, 8). Many active foci of ACL are known in Iran including Tehran, Kerman and Bam in the southeast, Shiraz in the south, Mashhad, Neishabur and Sabzevar in the north-east, Kashan, Yazd and parts of the Esfahan City, in the central parts (9, 10).

*Phlebotomus sergenti* is distributed in southern Mediterranean (Morocco, Algeria and Tunisia), northern Mediterranean (Italy, Portugal, Spain, southern France, Cyprus, Turkey) and The Eastern Mediterranean such as Saudi Arabia, Afghanistan, Pakistan and Iran (1). Three morphotypes of *P. sergenti* are identified as A, B and C, with some intermediate forms in Iran (5).

To study sand flies in detail and different aspects, including *Leishmania* interactions, it is essential to establish laboratory colonies of sand flies. Till now different techniques have been used for colonization of sand fly colonies in laboratory conditions (11–13). Among some 900 known sand fly species (14), so far only a few number have been colonized in the insectary furthermore, only a few have been reared continuously for several generations to experimental studies (15). Studies showed that, sand fly species and even laboratory colonies from different geographical origin are different in biological and physiological characteristic, such as saliva composition, longevity, fecundity and so on (16, 17). Various species of sand flies are categorized as restricted or permissive vectors to *Leishmania* parasites transmission, e.g. *Phlebotomus papatasi* and *P. sergenti*, are termed as restricted which allow a single *Leishmania* species to be developed within their gut and transmitted, while the others seem to be permissive and support multiple *Leishmania* species (18). Natural promastigote infections have been reported for *P. sergenti* in two ACL foci of Iran (19, 20). Furthermore, *Leishmania tropica* infection of this species using PCR was reported in Shiraz City, south of Iran (21). Moreover, transmitting the *L. tropica* by *P. sergenti* has been confirmed in Morocco (22), Algeria (23) and Tunisia (12) as well. Establishing of *P. sergenti* in the insectary is essential for understanding its physiology, its interaction with *L. tropica* and human and epidemiology of ACL in the country.

A first attempt to rear sand flies was done during 1963–1964 in Iran but it was not successful and failed after three generations (24). For the first time a successful colonization of *P. papatasi* was achieved by Iranian researchers in 2007 (25). The aims of current study were to establish of *P. sergenti* for the first time in the country and study on life-cycle data including productivity and fecundity of the colony and the developmental time of the first 5 generations. Determining the appropriate animal to blood feeding and longevity of the adults in insectary conditions were evaluated as well.

## Materials and Methods

### Sand fly collection

Sand flies were collected using CDC Miniature Light Trap and aspirator from indoors (bedrooms, toilets and bathrooms) and outdoors of Dehbakri, a rural district 50km far from the city of Bam (29°03′14.2″N, 57°54′31.6″E), Bam County, Kerman, Iran in September through August 2014. The collected sand flies were transferred into a cloth cage holding in a stainless steel framework (35×35×35cm) and then were transferred in an appropriate condition to the Sand fly Insectary of School of Public Health, Tehran University of Medical Sciences, Tehran, Iran.

### Colony initiation

The gravid, semi-gravid and engorged female sand flies were transferred into individual pots made from polyester (5.5cm height, 4cm diameter) which were plastered inside with a layer of Paris poured and left till egg lying. The unfed sand flies were transferred into a suspended nylon cloth cage and offered them a blood meal. Female sand flies were fed on a variety of animals including chicken, golden hamster or mouse. To improve the blood engorgement of females, males were also transferred into the cages and were covered with a dark cloth during blood feeding. After blood feeding, engorged females left undisturbed for 12–24h. Before transferring the blood fed sand flies, the plaster in the bottom of the pots were moistened with distilled water to encourage the sand flies to lay eggs on its moisten surface. To provide sufficient energy during egg incubation, the solutions of 20% sucrose and 50% honey syrup were offered for transferred sand flies till egg laying (26). Pots were kept in plastic boxes with a layer of moist fine soil in the bottom. High humidity (70–80%) in the boxes were prepared by moistened the soil in the bottom of the boxes with distilled water. The optimum temperature was adjusted to 26–28 °C. Pots were checked for oviposition daily and the female sand flies which laid eggs were removed to prevent development of fungal contamination. After oviposition, each female was mounted in Puri’s medium (27) and identified after 48–72h using the related morphological keys (28, 29). After identification, only *P. sergenti* was included and the other species were excluded from the study.

### Colony rearing

Sand fly colony was maintained in an isolated room with controlled temperature (26–28 °C) and 14:10 (light: dark) photoperiod based on modified method of Modi and Tesh (1983) (13). High humidity (70–80%) was provided by wrapping cages in plastic bags with hanging wet cloth inside. For larval rearing we used pots made from polyester (4.5cm height, 8.5cm diameter) with a big hole in the bottom. The pots bottom was filled with a 1cm thick layer of white plaster of Paris. Wet plaster provided humidity and a resting surface inside the pots during incubation period. The pots were covered by fine mesh gauze and engorged female sand flies along with males were transferred through a small hole in the gauze and the hole was then closed with a cotton wool pad.

Larval diet was a composted mixture of rabbit faces and rabbit pellets which mixed in equal amount in distilled water and left to fermentation in the room temperature. The prepared mixture was mixed daily to improve fermentation process. After completing the fermentation process resulting mixture was spread on special trays to dry. The dried mixture was ground and used to feed larval stages. The fermentation process normally lasts about 6 months, but in an innovative work, by adding yeast, fermentation process shortened to a few weeks (30). Pots contained larvae were put in plastic boxes which the bottom filled with fine sand moisten with distilled water. Emerged sand flies were released from pots into suspended net cages supported with a steel frame, wrapped in nylon bags. To adults feeding, soaked piece of cotton wool in 20% sugar solution and 50% honey syrup prepared for adult inside the cages. Adults were offered a blood meal twice a week. Blood fed females were kept undisturbed in net cages for 24h and then the engorged sand flies were transferred into the pots for oviposition. Almost one week after transferring, most females laid eggs and died. After 50% egg hatching, 2–3mm soil collected from gerbil colonies and some larvae food add into the pots. Interval shaking the soil layer prevents fungal growth on the bottom of the pots.

### Determining the appropriate host for blood feeding

To identify which animal was more appropriate as a blood source for *P. sergenti*, there conventional blood source in insectary which were available, easy to handle and housing, including chicken, golden hamster and BALB/c mice were selected. Five to seven days old sand flies were chosen to conduct the test. The experiment was done in 6 replication and 12 to 30 sand flies in the same generations (F4 to F6), were included in each one. Homogenous number of sand flies was transferred into cloth cage holding in a steel framework.

To improve the engorgement rate, males were transferred to cages as well. Before exposing to sand flies, animals were anesthetized using Ketamin hydrichloride (60mg per kg body weight) and Xylazine (15mg per kg body weight) for 1h. Since sand flies prefer blood feeding in dark, cages were covered by dark tissue. After 1h the numbers of blood fed and unfed sand flies in each cage counted and compared. Animal experimental protocol was approved by the Ethics Committee of Tehran University of Medical Sciences, Tehran, Iran (Protocol number: IR.TUMS.VCR.REC.1395.253).

### Life parameters assessment

Generations of F1 to F5 were used to study biological parameters of *P. sergenti*. For each generation 40 to 50 engorged *P. seregenti* females were included to assessing life parameters. New emerged sand flies were transferred into cloth cage holding in a steel framework with 20% sugar solution and 50% honey syrup. After 4 to 7 days sand flies were offered a blood meal on golden hamster. For all generations the source of blood feeding was the same. Adult sand flies were left hungry 12h before blood feeding. Engorged sand flies were kept undisturbed for 24h and then were transferred individually into a plastered line pot. All the individual pots were checked daily and information including time of blood digestion and mortality were recorded. The female sand flies were followed up until egg lying and were dissected after natural death. The number of laid and retained eggs was counted, recorded and fecundity was calculated for each individual sand fly. Fecundity defined as follow:

$$\text{Fecundity}=\frac{\text{No}\;\text{of}\;\text{laid}\;\text{eggs}\;\text{by}\;\text{each}\;\text{sand}\;\text{fly}}{\text{No}\;\text{of}\;\text{laid}\;\text{eggs}+\text{No}\;\text{of}\;\text{retained}\;\text{eggs}}\times 100$$

Natural death time for females and males was recorded. Main procedures during blood feeding to the adult stage and the production rate were assessed as well. The minimum developmental times (ranges) were recorded by observation of the first appearance of each stage in the rearing pots. The productivity was defined as the percentage of eggs from each generation which reached to the adult stage (31). To achieve this purpose, blood fed sand flies left to lay eggs individually. Laid eggs were counted and were followed up to hatch. The first egg hatching was recorded, and at the following the major interval times including: L4-pupa and pupa-adult were recorded as well. Emerged adults sand flies including males and females counted and recorded.

### Statistical analysis

Statistical analysis was performed using SPSS16. One-Way ANOVA, *X^2^* and Duncan (P= 0.05) tests were employed to compare different parameters in five generations.

## Results

The reared species identified as *P. sergenti.* Blood feeding percentage of *P. sergenti* on chicken, BALB/c and golden hamster were evaluated. The results indicated that *P. sergenti* has higher blood feeding percentage on chicken compared to BALB/c and golden hamster in the same condition. Of 300 sand flies (6 replications), 170 took a blood meal (56.6%) which 72 (42%), 36 (21%) and 62 (37%) individuals took blood on chicken, BALB/c and golden hamster, respectively. Statistical analysis (*X^2^* test) showed there is no significant difference (P= 0.388) in blood feeding percentage between chicken and golden hamster, on the other hand the blood feeding percentage between chicken or golden hamster and BALB/c was statistically significant (P= 0.001).

The average optimum time for blood feeding was 5.6 (range: 4.5–6.8) days after emerging. The average time for blood digestion was 3.4 days and the maximum and minimum time for egg incubation (The time which was needed to mature eggs inside uterus) were 10.9 days in F1 and 7.1 days in F5 after blood feeding. The time of the egg hatching (1^st^ instar larva) varied from 14.3 days in F2 and F5 to 17.6 days in F1, however, the mean time was 15 days after blood feeding and the total developmental time for larval stages recorded at 18.3 days (range: 16.1–20.5). The time of pupa formation varied, and the larvae pupated at the maximum and minimum time of 36 in F1 and 30.6 in F3 and at average 33.3 days after blood feeding. Diapause was occurred in some portion of the L_4_ larvae which lasted two month to over one year, and the recorded times was based on the minimum time for pupa appearing. The total developmental time for *P. segenti* adult was 41.2 days in average (range: 38.9–44.1) after blood feeding (Table 1).

**Table 1.** Minimum developmental time of *Phlebotomus sergenti* (days after blood feeding) over five generation

Generations	Optimum blood feeding time	Blood digestion	Egg incubation	1^st^ instar larva	Pupa	Adult
**F1**	5.7±1.4	3.2±0.4	10.9±4.3	17.6±2.1	36±5.6	44.1±6.8
**F2**	5±1.1	3.5±0.5	8.2±2.8	14.3±1.3	32.2±5.3	40.8±5.2
**F3**	6.8±1	3.9±0.4	8.5±2.8	14.5±1	30.6±3	38.9±3.9
**F4**	4.5±0.9	3±0.3	9.2±3.7	14.7±1.2	35.2±6.5	41.2±6.5
**F5**	6.8±1.1	3.4±0.4	7.1±2.1	14.3±2.6	32.9±3.2	41.3±3.6
**Total average**	5.6 ±1.2	3.4±0.5	8.7±3.6	15±1.6	33.3±4.2	41.2±4.6

**Fig 1.**
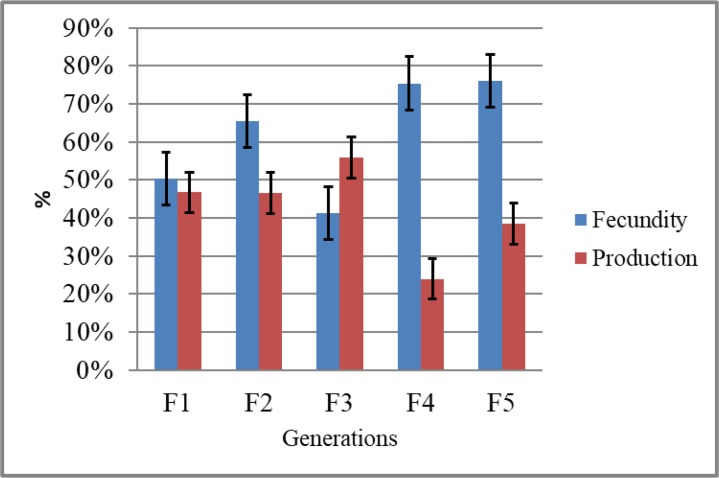
Comparison of the fecundity and production rates of *Phlebotomus sergenti* over five generations

The average number of laid eggs from F1 to F5 generations showed an ascending trend and the average number of laid eggs was 55.1 per female. It is interesting to point that, the maximum and minimum egg numbers ever seen during this study were 120 in F2 and one egg in F1 and F4 per female, respectively. The maximum and the minimum number of retained eggs were 49.7 in F1 and 21.5 in F4. As Table 2 shows, fecundity has ascending trend from F1 to F5 generations and the average calculated fecundity for all generations was 61.6% (range: 41.1–76.1%). The maximum and minimum longevity of males were 18.3 and 13.1 days in F1 and F3, respectively, in insectary condition, while the average longevity of females was longer (15.2 days for females vs. 14.8 days for males). As Table 2 shows longevity has an approximate descending trend either for females or males from F1 to F5 generations. The average number of emerged males (15.1) was more than females (11.3) per egg batches. To assess the efficacy of the rearing procedure, production (yield) of each generation was calculated. The maximum and minimum production were obtained from F3 (55.8%) and F4 (23.9%) and the average production was 42.2%. Except for the time of blood digestion (P> 0.05), the statistical analysis (One-Way ANOVA) showed significant differences (P< 0.001) in other recorded or calculated parameters among five generations. In Fig. 1 two important ecologic parameters of *P. sergenti* including fecundity and production rate are compared. Surprisingly, as chart shows, during F1 to F5 as fecundity increased, production decreased correspondingly.

**Table 2.** Some biological parameters of *Phlebotomus sergenti* over five generations

**Generations**	**No. of Egg laid**	**No. of Retained egg**	**Fecundity**	**Female longevity**	**Male longevity**	**Average No of Female**	**Average No of Male**	**Production rate**
**F1**	48.2±3.8	49.7±3.6	50.2%	17.7±4.7	18.3±4.7	10.8±7.7	14.2±2.1	46.7%
**F2**	48±3	25.8±2.7	65.3%	14±3.7	13.8±3.7	12.8±7.6	16.6±1.1	46.5%
**F3**	33.6±2.2	48±2.7	41.1%	14.8±3.2	13.1±3.7	13.9±6.6	18.8±9.2	55.8%
**F4**	71±2.8	21.5±2.5	75.4%	15.9±3.8	15.2±4.3	5.5±3	9.6±2.8	23.9%
**F5**	74.8±1.9	30±1.7	76.1%	13.7±2	13.8±2.3	13.6±7.1	16.4±7.6	38.4%
**Total average**	55.1±3.2	35±2.9	61.6%	15.2±4	14.8±4.3	11.3±7.8	15.1±9.8	42.2%

During this study diapause phenomenon of *P. sergenti* was checked as well. Observation showed that, diapause occurred in L_4_ and lasts two months to over one year. Furthermore, the results indicated that, this phenomenon mostly occurred in F2 and F5 generations.

## Discussion

Establishment of sand fly laboratory colonies and their biological parameters give a detailed insight into their life cycle and provides an opportunity to study on parasite and invertebrate host interaction, susceptibility to insecticides and, etc, this information pave the way to control sand fly borne diseases. In the current study, *P. sergenti* was colonized for the first time in the country, and the biological parameters were investigated as well.

The results of the blood feeding percentage test showed that, chicken is more preferred by *P. sergenti*, then golden hamster and then mouse. Although feeding percentage on chicken was more than golden hamster and BALB/c but due to difficulties in handling and anesthetizing of chicken, golden hamster was used to routine blood feeding. The reason might be that chicken in comparison with several avian and mammalian species showed the lowest defensive behavior against blood feeding (32). The results of a study conducted in the field suggested that the local population of *P. sergenti* is highly ornithophilic (33). One study showed that chickens and human are relatively unattractive hosts for blood feeding of *Lu. Longipalpis*, however, cows and pigs were preferred (34). Conversely, chickens are more attractive hosts for *Lutzomyia longipalpis* in locations that domestic animals are absent or in low numbers (35).

Optimum time for blood feeding recorded in average of 5.6 days which is affected mainly by species, age, temperature, humidity and host availability (36). In this study average time for blood digestion was 3.4 days after blood feeding on golden hamster in 26–28 °C temperature, this time were recorded for *Lutzomyia evansi* as 3.5 (range: 2–5) days (37). Required time for blood digestion varied and affected by temperature (36) and blood source (38). So that, time of blood digestion was the only parameter which was not statistically different among the 5 generations. The average time for egg maturation recorded to be 8.7 (range: 7.1–10.9) days, the same time in a study completed on *P. sergenti* originated from Turkey showed to be 8.1 days (range: 7–9). The maximum and minimum time of egg maturation for *P. papatasi* was recorded at 12.4 in F1 and 5.6 days in F5 (39), and in another study the maximum and minimum time for *Phlebotomus perniciosus* was shown to be 10 in F4 and 5.5 days in F1 (31). It seems that, from F1 to F5 *P. sergenti* needed less time to mature its eggs (F1: 10.9, F5: 7.1 days), and similar phenomenon was seen in *P. papatasi* as well (39). The reason might be due to adaptation process in insectary condition. This time was reported to be 9 days for *Phlebotomus martini*, 7 days for *Sergentomyia schwetzi*, and *Sergentomyia africana* (40). The average time for 1^st^ instar larva appearing (egg hatching) was 15 days after blood feeding and the same time for *P. sergenti* originated from Turkey recorded at 14.2 (range: 12–18) days after blood feeding (15). For *P. perniciosus* this time was recorded at minimum 5.6 (F1) and maximum 7 days (F10) after pre-oviposition period (31). In the current study total developmental time of larval stages was 18.3 days (range: 16.1–20.5) which was reported to be 18 to 22 days for *P. perniciosus* (31). The formation of pupa varied, as some portion of the L4 larvae might go to diapauses (2 month to over one year), the recorded times were based on the minimum time for pupa emerging. As it is shown, lengthy time of diapause phenomenon makes the colony maintenance difficult in some species including *P. sergenti*, *P. ariasi*, *P. perfiliewi* and *P. simici* (15). The average time for pupa emerging was 33.3 (range: 30.6–36) days after blood feeding and the development time of pupa was recorded at 7.8 days (range: 6–8.6). This time for *P. papatasi* was minimum 7.4 to maximum 9.3 days in the first 5 generation (39). In the current study an average time for adult emerging recorded at 41.2 (range: 38.9–44.1) days after blood feeding. The same time was 40.3 (range: 38–45) days for *P. sergenti* originated from Turkey (15), for *P. papatasi* was 35.4 days (range: 31.5–42.7) (39) and for *Lu. evansi* and *P. perniciosus* were 41.8 and 42 days, respectively (31, 37, 40). The mean developmental time from egg to adult recorded at 47.2 to 52.6 days for *P. papatasi* in Iran (25).

As the results showed, the number of laid eggs followed an increasing trend which it could be a sign of adaptation through generation. The same phenomenon was shown in another study which the average minimum and maximum numbers of laid eggs obtained as 34.3 in wild-caught and 55 in F10 for *P. perniciosus* (31). The average number of laid eggs was 55.1per female in this study which was much more than the number obtained from *Lutzomyia sanguinarms*, *Lu. gomezi*, *Lu. trapidoi*, *Lu. ylephilctor*, *Lu. panamensis* and *Lu. pessoana* that reported as 27, 29, 21, 27, 28 and 20 respectively (11). The maximum number of eggs ever seen during this study was 120 per female in F2, this number was 112 for *P. papatasi* and 85 for *P. perniciosus* (31, 39). As the results showed, longevity was being decreased over 5 generations either in females or males which maybe the result of inbreeding that affected biological fitness of the colonies. The number of emerged males was more than females and the average longevity of blood fed females was longer than males.

As the results showed, the minimum and maximum of productivity were 23.9% and 55.8% in F4 and F3 respectively. These rates reported as 8.5% in F7 and 56.1% in F3 for *P. papatasi*, (25) and 9.9% in F4 and 47.3% in F6 for *P. perniciosus* (31). In another study, from F1 through F6, the average productivity was recorded at 44.08% (range: 29.6 to 55.2 %) for *P. papatasi* (39). As mentioned above the number of laid eggs increased from F1 to F5, it is expected that the production rate will increase consequently, however, it decreased. The reason behind this incompatibility could be the reduction of biological fitness or population bottleneck (or genetic bottleneck).

Due to changes which occurred during adaptation process, almost all biological parameters that recorded were statistically different among five generation. F4 is the significant point of the adaptation as it was shown by other study (31). During the establishing of this colony we faced a lot of difficulties and obstacles including high mortality during the immature phases due to several reasons including fungal, bacterial and mite infection which feed on eggs and young larvae, low blood preference specially in the early generations and mortality of engorged females before egg laying. To prevent mite infection the inner wall of the boxes containing pots were impregnated with petroleum jelly or liquid glue. Also, we add a layer of fine soil collected from vicinity of gerbils’ colony, in the bottom of larval pots. Interval shaking the soil layer prevents fungal growth.

## Conclusion

It is concluded that, colony of *P*. *sergenti* has been established for the first time in Iran and the biological parameters of this species was studied in detail. Average interval time from egg to 1^st^ instar larva, pupa and adult of the species recorded at 6.3, 24.6 and 32.5 days respectively. For colony initiation, chicken is recommended as a blood source but due to difficulties in handling and anesthetizing of chicken, golden hamster was suggested to routine blood feeding of *P*. *sergenti.* Establishment of this colony and defining its biological parameters in detail provides an opportunity for scientists to conduct research on biology, sand fly-parasite interaction, susceptibility to insecticides, transgenic and paratransgenesis experiments on this species.
